# Draft Genome Sequence of the Pathogenic Oomycete *Pythium insidiosum* Strain Pi-S, Isolated from a Patient with Pythiosis

**DOI:** 10.1128/genomeA.00574-15

**Published:** 2015-06-18

**Authors:** Thidarat Rujirawat, Preecha Patumcharoenpol, Tassanee Lohnoo, Wanta Yingyong, Tassanee Lerksuthirat, Sithichoke Tangphatsornruang, Prapat Suriyaphol, Laura J. Grenville-Briggs, Gagan Garg, Weerayuth Kittichotirat, Theerapong Krajaejun

**Affiliations:** a Department of Pathology, Faculty of Medicine, Ramathibodi Hospital, Mahidol University, Bangkok, Thailand; b Systems Biology and Bioinformatics Research Group, Pilot Plant Development and Training Institute, King Mongkut’s University of Technology Thonburi, Bangkok, Thailand; c Research Center, Faculty of Medicine, Ramathibodi Hospital, Mahidol University, Bangkok, Thailand; d Molecular Medicine Program, Multidisciplinary Unit, Faculty of Science, Mahidol University, Bangkok, Thailand; e Genomic Research Laboratory, National Center for Genetic Engineering and Biotechnology, National Science and Technology Development Agency, Pathumthani, Thailand; f Bioinformatics and Data Management for Research, Office for Research and Development, Faculty of Medicine, Siriraj Hospital, Mahidol University, Bangkok, Thailand; g Department of Plant Protection Biology, Swedish University of Agricultural Sciences, Alnarp, Sweden; h CSIRO Agriculture Flagship, Centre for Environment and Life Sciences, Floreat, Western Australia, Australia

## Abstract

*Pythium insidiosum* is an oomycete that causes a life-threatening infectious disease called pythiosis in humans and animals living in tropical and subtropical countries. Here, we report the first draft genome sequence of *P. insidiosum*. The genome of *P. insidiosum* is 53.2 Mb and contains 14,962 open reading frames.

## GENOME ANNOUNCEMENT

Morphologically, oomycetes look like fungi, but they separately and independently evolved from the metazoan ancestor into a group of eukaryotic microorganisms that have unique genetic, biochemical, and physiological characteristics (1, 2). While almost all pathogenic oomycetes infect plants, the oomycete *Pythium insidiosum* is capable of infecting humans and other animals living in tropical and subtropical areas of the world and causes a life-threatening infection called pythiosis (1, 3, 4). Surgical removal of an infected organ (eye or leg) or death is usually the final clinical outcome for patients with pythiosis (4). How evolution contributed to virulence and pathogenicity of *P. insidiosum* needs to be explored at the genome level.

Availability of next-generation sequencing technologies provides opportunities to sequence whole genomes of nonmodel organisms, including *P. insidiosum*. Here, we report the first draft genome sequence of *P. insidiosum*. The conventional extraction protocol (5) was used to extract genomic DNA (gDNA) of the *P. insidiosum* strain Pi-S, which was isolated from a Thai patient with vascular pythiosis (the most common form of *P. insidiosum* infection). Identity of the organism was confirmed by culture analysis and rDNA sequence. The extracted gDNA underwent whole-genome sequencing using a combination of the Illumina HiSeq2000 platform by Yourgene Bioscience, Taiwan (http://www.yourgene.com.tw), and the 454 Genome Sequencer FLX Titanium platform by the National Center for Genetic Engineering and Biotechnology, Thailand (http://www.biotec.or.th). For one library, the 454 platform provided 342,637 raw reads and 200,682,697 raw bases, which were assembled by Newbler version 2.8 (Roche) to the total contig length of 24,383,569 bases (number of contigs, 14,455; average contig length, 1,686). The Illumina platform was used to sequence one paired-end (180-bp insert) and three mate pair (5-kb, 8-kb, and 15-kb insert) libraries. After sequences were quality trimmed by CLC Genomics Workbench (http://www.clcbio.com), all Illumina libraries yielded a total of 240,448,339 reads and 22,926,078,291 bases (average read length, 95), which were then assembled by ALLPATHS-LG version 44588 (6) to the total contig length of 52,806,015 bases (number of contigs, 1,261; average contig length, 41,876). Gaps were filled using GapCloser (7). Merging the 454- and Illumina-derived assembled sequences, using Minimus (8), provided the draft genome sequence of 53,239,050 bases, comprising 1,192 contigs (average contig length, 44,664 [range, 966 to 655,053]; *N*_50_, 146,252), with a G+C content of 52% and an N composition of 10%.

The draft genome was assessed to be 92% complete, using CEGMA with a well-defined set of 248 highly conserved genes from eukaryotes (9, 10). Transcript mapping (11) and gene prediction by MAKER2 (12) revealed 14,962 open reading frames (ORFs), of which 3,579, 1,254, and 10,129 ORFs were predicted by both transcript mapping and gene prediction, only transcript mapping, or only gene prediction, respectively. A BLASTp search against the NCBI nonredundant protein database found significant matches (*E* value, less than −6) in 89.7% of ORF-translated proteins.

### Nucleotide sequence accession numbers.

Related genome sequence data of *P. insidiosum* strain Pi-S has been deposited in the DDBJ under the accession numbers BBXB01000001 to BBXB01001192.
